# Supplementary material for: The Potential for High-Priority Care Based on Pain Through Facial Expression Detection with Patients Experiencing Chest Pain
Source: Diagnostics (Basel). 2024 Dec 25;15(1):17. doi: 10.3390/diagnostics15010017 (PMC11720015; doi:10.3390/diagnostics15010017)

Supplementary Material

"The Potential for High-Priority Care based on Pain through Facial Expression Detection with Patients Experiencing Chest Pain"

```
#Pseudocode object detection:
Backbone = EfficientRep(
num_repeats=[1, 12, 12, 18, 6],
out_channels=[64, 128, 256, 512, 1024] )

Neck = RepPAN(
num_repeats=[12, 12, 12, 12],
out_channels=[256, 128, 128, 256, 256, 512] )

Head = EffiDeHead(
in_channels=[128, 256, 512],
num_layers=3,
begin_indices=24,
anchors=1,
out_indices=[17, 20, 23],
strides=[8, 16, 32],
iou_type='ciou' )

Model = {
"backbone": Backbone,
"neck": Neck,
"head": Head }

input_image = preprocess(image)
features = Backbone.extract_features(input_image)
aggregated_features = Neck.aggregate(features)
detections = Head.detect(aggregated_features)
final_results = post_process(detections)
```

Figure S1. Proposed model pseudocode

Table S1. YOLOv4 model and YOLOv6 model optimization configuration

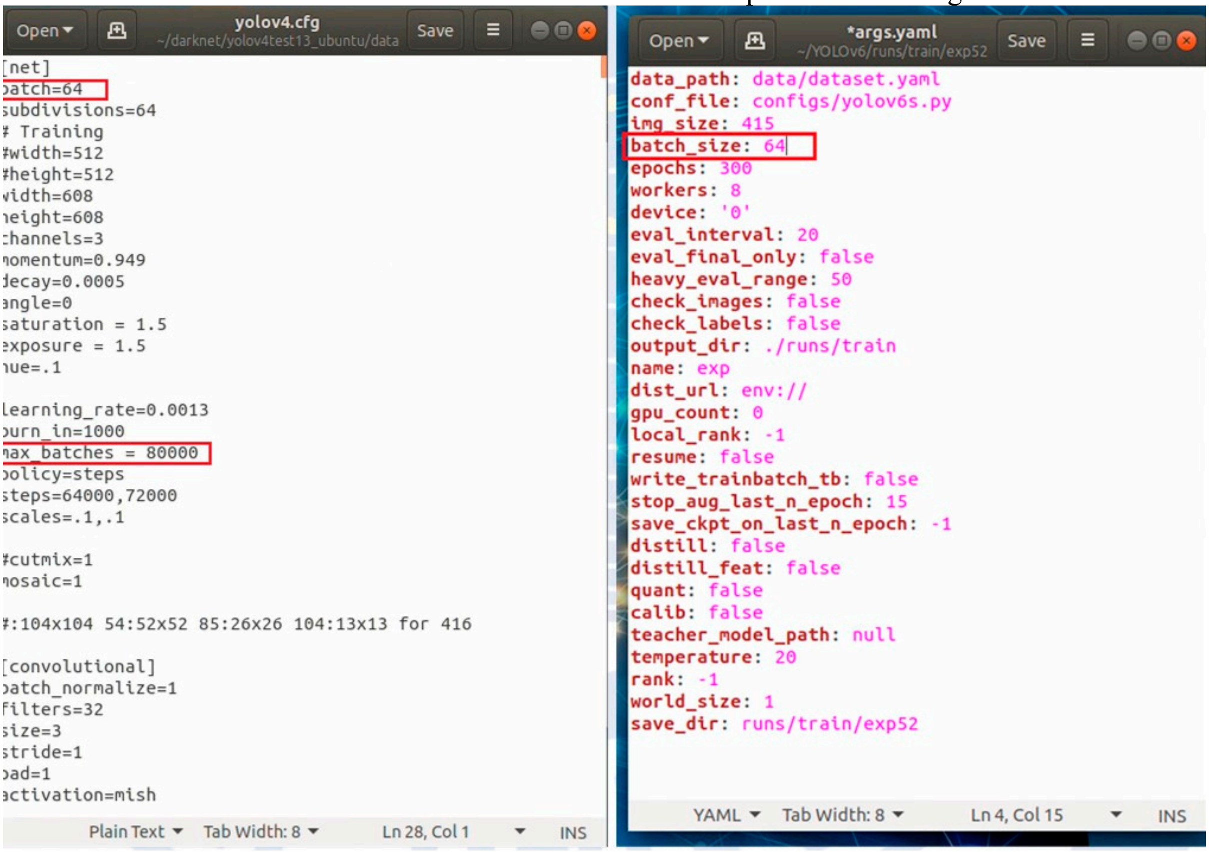

Supplement: Supplementary file 1 [file diagnostics-15-00017-s001.zip › diagnostics-3277028-supplementary.pdf]
